# Case 1/2019 - A 51-year-old Man with Arterial Hypertension, Aortic Dissection and Aortic Valve Regurgitation, in Addition to Heart Failure with Unchanged Clinical Course After Surgical Intervention

**DOI:** 10.5935/abc.20190013

**Published:** 2019-02

**Authors:** Desiderio Favarato, Vera Demarchi Aiello

**Affiliations:** Instituto do Coração (Incor) - Hospital das Clínicas da Faculdade de Medicina da Universidade de São Paulo (HC-FMUSP), São Paulo, SP - Brazil

**Keywords:** Hypertension, Aortic Valve Insufficiency, Aortic Aneurysm/surgery, Aneurysm, Dissecting/surgery, Heart Failure

A 51-year-old male, hypertensive patient, a former smoker, was transferred for treatment of thoracic aortic dissection and heart failure.

After a three-week period with progressively more intense chest pain, accompanied by dyspnea, sweating and vomiting, he was admitted to the Hospital in the city where he lived.

At hospital admission, he had high blood pressure and the diagnosis of thoracic aortic dissection was made. He received antihypertensive and beta-blocker medications. On the fifth day, he was transferred to Instituto do Coração for treatment. At that moment, he was asymptomatic.

The physical examination (June 14, 2012) showed good general health status, paleness, ++ / 4+, increased jugular venous pressure, heart rate of 80 bpm, blood pressure 80 x 60 mmHg; clear lungs; cardiac auscultation disclosed rhythmic heart sounds and ++++ diastolic murmur on the left sternal border, no abdominal alterations, lower limbs without edema, besides palpable and symmetrical pulses.

Laboratory tests (June 14, 2012) showed hemoglobin 15.9 g/dL; hematocrit, 49%; leukocytes, 10,080/mm^3^ (neutrophils 64%, eosinophils 7%, lymphocytes 21%, and monocytes 8%); platelets 232,000/mm^3^; CKMB, 1.33 ng/mL; troponin I, 0.106 ng/mL; urea, 38 mg / dL; creatinine, 0.94 mg /dL; sodium, 137 mEq/L; potassium, 4.2 mEq/L; prpthrombin time (PT) (INR), 1.1; APTT time ratio, 0.78; AST, 30 U/L; ALT, 61 U/L; gamma-GT, 116 IU/L; alkaline phosphatase, 81 U/L; and negative serology for hepatitis B, C, and HIV.

The electrocardiogram (ECG) performed on June 16, 2012 showed sinus rhythm, left atrial and left ventricular overload with strain pattern (Figure 1).

**Figure 1:**
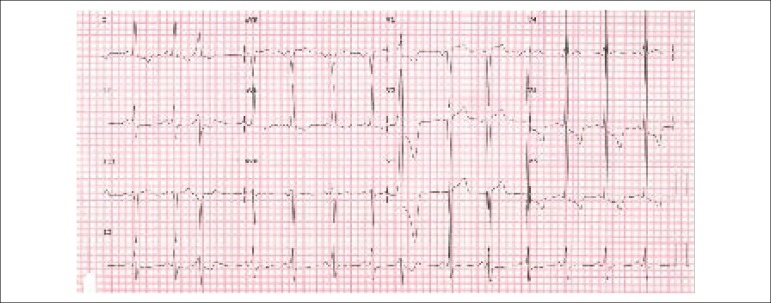
ECG showing atrial and left ventricular overload, the latter with a strain pattern.

The echocardiogram performed on June 17, 2012, disclosed the following measurements: aorta, 37 mm; left atrium, 48 mm; septal thickness and posterior wall, 9 mm; left ventricle, 87/78 mm; ejection fraction, 22%. The patient showed eccentric hypertrophy with diffuse hypokinesis; moderate mitral regurgitation; marked aortic regurgitation; ascending aortic dissection was observed, with the original intimal tear 25 mm from the valve plane. The aortic measurements at the different levels were: aortic sinus, 37 mm, sinotubular junction, 46 mm, ascending aorta, 67 mm and aortic arch, 34 mm.

The posteroanterior chest X-ray performed on June 18, 2012 showed normal lung fields, aorta with an image suggestive of aneurysm, and enlarged cardiac area (Figure 2)

**Figure 2:**
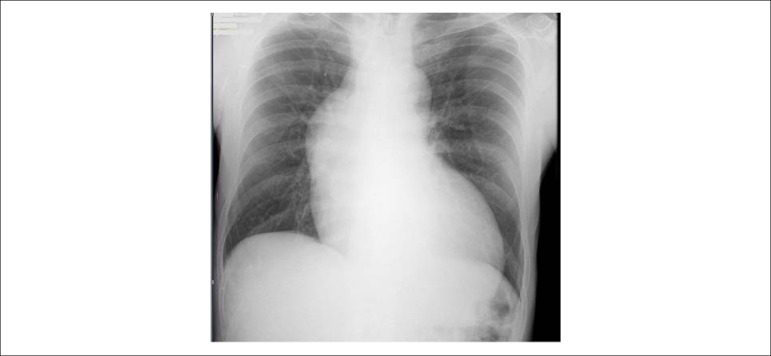
Chest x-ray: mediastinal enlargement, suggestive of aortic aneurysm and cardiomegaly.

The coronary angiography did not show any coronary lesions. The pressures were: aorta: (syst/diast/mean) 100/50/67 mmHg and left ventricular: (Syst/initial diast/end diast) 100/10/20 mmHg. The left ventricle showed diffuse hypokinesis. There was marked aortic regurgitation and an ascending aortic aneurysm, with an image suggestive of dissection (Figure 3).

**Figure 3:**
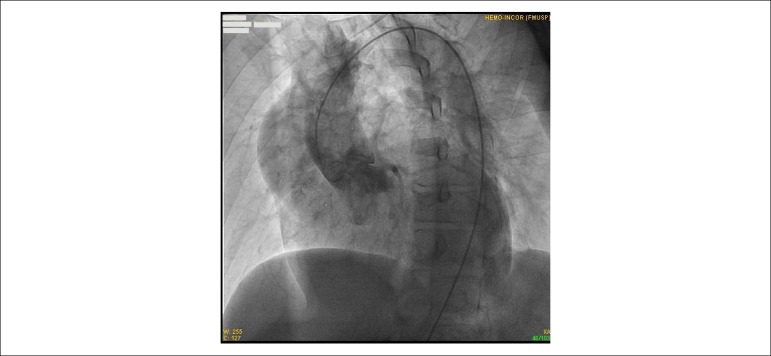
Aortography. Ascending aortic aneurysm with dissection and catheter in the true lumen.

The patient underwent surgery for repair of the ascending aortic dissection, with the interposition of a Dacron tube and aortic valve repair (June 19, 2012). The postoperative period was uneventful, and the patient was discharged on the ninth postoperative day.

Almost a month after hospital discharge (July 11, 2012), he sought emergency medical attention for worsening of dyspnea, attributed to non-adherence to the prescribed medication.

The physical examination (July 11, 2012) disclosed a heart rate of 60 bpm, blood pressure 80 x 60 mmHg; clear lungs; normal cardiac auscultation, no abdominal alterations; lower limbs without edema and no signs of deep vein thrombosis, with normal pulses.

The laboratory tests (July 18, 2012) showed hemoglobin 10.7 g/dL; hematocrit, 32%; leukocytes, 9,750/mm^3^ (band cells 1%, segmented 69%, eosinophils 8%, basophils 3%, lymphocytes 14%, monocytes 5%), platelets, 443,000/mm^3^, C-reactive protein, 65.05 mg/L; urea 29 mg/dL; creatinine, 0.90 mg/dL, sodium, 130 mEq/L; potassium, 4.8 mEq/L; magnesium, 1.70 mEq/L, BNP, 1280 pp / mL, venous lactate 21 mg/dL; venous gasometry: pH 7.38, pCO_2_, 48 mmHg; pO_2_, 34.9 mmHg, O_2_ saturation, 53.8%; bicarbonate, 26.5 mEq/L, base excess, 2 mEq/L. Blood, urine and catheter tip cultures were negative.

One month after this episode he was again brought to emergency care (August 11, 2012) with dyspnea on minimal exertion, orthopnea and oliguria for two days. He denied coughing, fever, coryza or diarrhea; chest pain or palpitation. He reported correct use of medications and hydrosaline restriction.

The physical examination on admission showed blood pressure of 80x60 mmHg, heart rate of 102 bpm, fine pulses and decreased peripheral perfusion; jugular venous pulse was present; rhythmic heart sounds, presence of third heart sound, mitral systolic murmur +++ / 6+, tricuspid systolic murmur +++ / 6+ and aortic systolic murmur ++ / 6+; abdominal assessment - liver palpable at 5 cm from the right costal border; palpable and symmetrical lower-limb pulses.

He was started on intravenous dobutamine, vasodilator and diuretic drugs, in addition to the antibiotics vancomycin and meropenem.

Laboratory tests (August 11, 2012) showed hemoglobin: 9.2 g/dL, hematocrit: 31%, leukocytes: 12,980/mm^3^ (neutrophils 74%, eosinophils 1%, basophils 1%, lymphocytes 19%, monocytes 5%), platelets: 395,000/mm³, C-reactive protein: 79.58 mg/dL, urea: 55 mg/dL, creatinine: 1.26 mg/dL, sodium: 135 mEq/L, potassium: 5.1 mEq/L, prothrombin time: (INR) = 1.2, APTT:(rel) = 0.97, venous lactate: 55 mg/dL

The chest x-ray showed pulmonary congestion and cardiomegaly.

The echocardiogram (August 13, 2012) showed the following measurements: aorta, 41 mm; left atrium, 50 mm; right ventricle, 35 mm; septum, 9 mm; posterior wall, 10 mm; left ventricle, 86 mm; ejection fraction, 20%; hypertrophic left ventricle with diffuse hypokinesis; right ventricle with moderate hypokinesis; severe mitral regurgitation; normal aortic valve; pulmonary valve with indirect signs of pulmonary hypertension; estimated pressure of 43 mmHg and presence of Dacron tube in the aorta.

The transesophageal echocardiogram (August 16, 2012) also showed the presence of marked tricuspid regurgitation and no images suggestive of thrombi or vegetations were observed.

The laboratory tests performed on August 12 showed hemoglobin decrease to 7.7 g/dL and no evidence of bleeding and, therefore, an upper digestive endoscopy was requested, which did not show any alterations.

A Doppler ultrasonography of the lower limbs carried out due to suspicion of pulmonary thromboembolism (recent RV dysfunction) showed no signs of thrombosis. The patient then underwent a chest tomography, which identified a small consolidation on the right side, with right pleural effusion.

He developed refractory shock, despite the use of antibiotics. The right pleural effusion was punctured, and the presence of transudate was identified.

All blood cultures and urine cultures were negative. He required orotracheal intubation for ventilatory support on August 29, 2012, developing an increasing need for noradrenaline, and the antibiotics were replaced by daptomycin, micafungin, and rifampicin.

Laboratory reassessment (September 4, 2012) showed: hemoglobin: 10 g/dL; hematocrit: 33%; leukocytes: 5,450/mm³ (neutrophils 85%, lymphocytes 10% and monocytes 5%); platelets: 313,000/mm³; C-reactive protein: 221.38 mg/L; urea: 44 mg/dL; creatinine: 2.21 mg/dL; phosphorus: 2.2 mg/dL; magnesium: 1.6 mEq/L.

The patient remained in shock and died (September 4, 2012).

## Clinical aspects

This was a male patient, who presented with ascending aortic dissection, and who, even after surgery for dissection repair, developed a picture of severe heart failure and died.

The International Registry of Acute Aortic Dissection (IRAD) showed that patients with aortic dissection were older than our patient, 61 years; and a majority of males (63%). Regarding the diseases related to the dissection, they were: Marfan syndrome (6.7%), hypertension (69.3%), atherosclerosis (24.4%), previously known aortic aneurysm (12.4%), previous aortic dissection 3.9%) and diabetes mellitus (4.3%). Also, 15.9% had a history of previous cardiac surgery and iatrogenic cause in 4.8% (1.7% coronary angiography and 3.1% after cardiac surgery).^1^ The 2015 update of the same registry, with a ten-fold higher number of patients, showed an increase in hypertension (75.5%) and a decrease in the presence of Marfan syndrome (4.5%), (75.5%), atherosclerosis (19.6%) and previous cardiac surgery (10.6%).^2^

Genetic tests can be performed in the presence of aortic aneurysm in younger patients. The syndromes related to the presence of aortic aneurysms are Marfan, Loeys-Dietz, Ehler-Danlos syndromes, the cutis laxa or elastolysis and that related to a defect in the transforming growth factor beta (TGFβ).

Classically, the genetic alterations found in Marfan syndrome are related to the fibrillin-1 gene.^3^ Changes in the physical examination involve ocular (myopia, ectopia lentis, and risk of retinal detachment), skeletal (exaggerated growth and joint laxity, exaggerated growth of the extremities) and cardiovascular alterations (dilation of the aorta at the level of the sinuses of Valsalva, predisposing to dissection). In the present case, we do not have a description of these phenotypic changes that might suggest such diagnosis.

Loeys-Dietz syndrome includes several manifestations similar to those of Marfan Syndrome, but they also include hypertelorism, broad or bifid uvula, cleft palate and generalized arterial tortuosity, aneurysms, and arterial dissection. Generally no increase of extremities and ocular alterations are observed.^4^

As well as for Marfan syndrome, we do not have evidence in the present case to suggest such a diagnosis.

The Ehler-Danlos syndrome is characterized by fragility of the connective tissue and the manifestations occur in the skin (hyperelasticity, atrophic scars and easy ecchymosis), joints (hypermotility, frequent dislocations and arthralgias) and vessels (aneurysms and spontaneous vessel ruptures). The vascular form, in which dissection occurs most frequently, occurs through a mutation in the alpha-1 gene of type III collagen, with a silent substitution that leads to the replacement of glycine in the collagen chain.^5^

We also have no clinical evidence of such changes in the present case. A more recent study showed gene panel alterations in 25% of patients with aortic aneurysms.^6^

What should be taken into account is that even today the criterion for the indication of surgical treatment for rupture prevention remains the diameter of the aneurysm, 50 mm.

In the present case, the patient had arterial hypertension and it must have had an important role in the development of the thoracic aneurysm and its rupture. Biomechanical studies show that for hypertension to lead an aneurysm development, there must be concomitant failure in the composition and maintenance of the extracellular matrix and membrane receptors. Consequently, there is damage to the mechanical stress transduction in the cell-signaling response.^7,8^

Regarding the patient’s unfavorable evolution after the surgery, it is probably due to the long evolution of the aortic aneurysm with aortic valve regurgitation, leading to left ventricular dilation and severe dysfunction, which in the very late states do not undergo regression or relief despite valve replacement surgery and progress to progressive heart failure. The ECG disclosed left ventricular overload with strain and the echocardiogram showed large left ventricular dilation and marked dysfunction.

The European Guidelines of Cardiology and Thoracic Surgery recommends valve replacement surgery in patients with aortic dilation or aortic regurgitation accentuated with symptoms. In asymptomatic patients, this recommendation appears if there is reduction in ejection fraction (<50%) or ventricular dilation (diastolic diameter >70 mm or diastolic >50 mm).^9^

The patient had degrees of dilation (diastolic diameter, 87 mm and systolic diameter, 78 mm) and ventricular dysfunction (ejection fraction of 22%) well beyond those recommended for the indication of valve replacement surgery.

This is the most plausible explanation for the poor evolution. **(Dr. Desiderio Favarato)**

**Diagnostic hypotheses:** Aneurysm in the thoracic aorta, chronic aortic valve regurgitation, aortic dissection. Etiology: arterial hypertension and extracellular matrix disease of the aorta. Final clinical picture: cardiogenic shock due to valvular heart disease. **(Dr. Desiderio Favarato)**

## Necropsy

**Figure 4:**
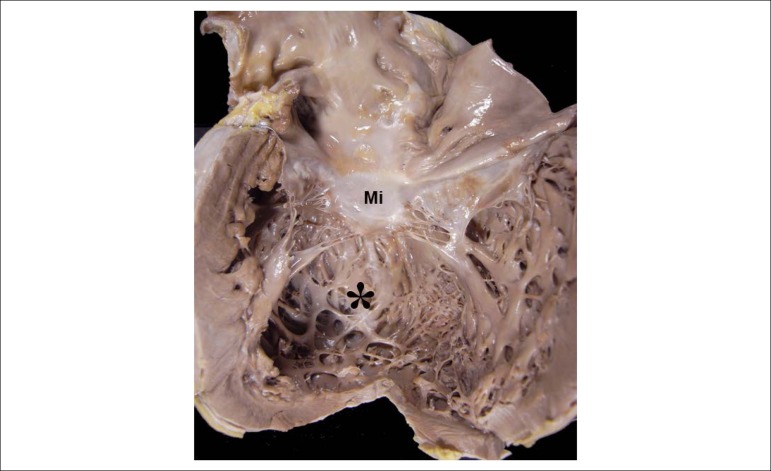
Macroscopic aspect of the open left heart chambers, with marked left ventricular dilation (asterisk); Mi: mitral valve.

**Figure 5:**
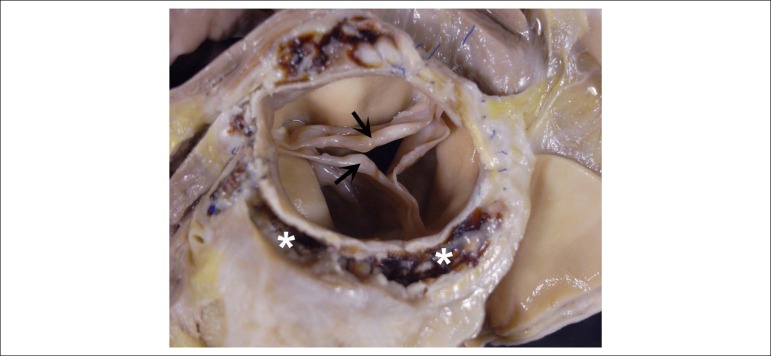
Aortic valve seen from its arterial aspect. There is thickening of the free edge of the semilunar leaflets (arrows) and lack of central coaptation. A multiloculated cavitary lesion (asterisks) is seen around the aorta, from which a pasty material emerged.

**Figure 6:**
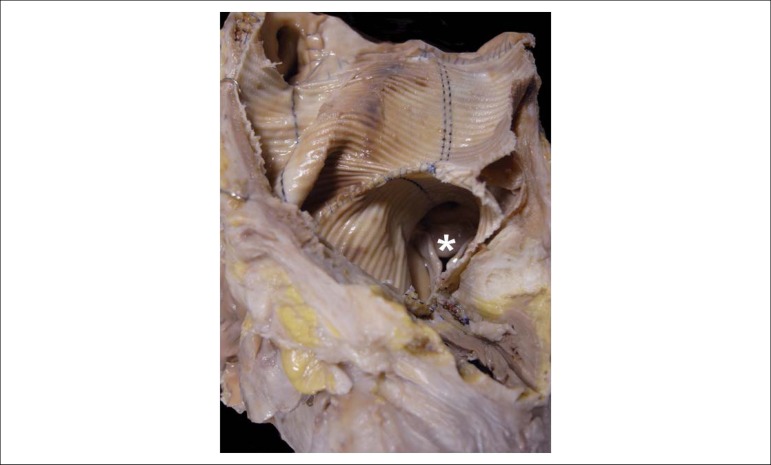
Macroscopic aspect of the corrugated synthetic tube that replaced the ascending aorta. The aortic valve can be seen in the background (asterisk).

The heart weighed 890g, with marked increase in volume and dilation of all chambers, predominantly the ventricles (Figure 4). The atrioventricular valves showed no abnormalities. The aortic valve showed thickening of the free margins of the semilunar leaflets, with a central non-coaptation aspect, compatible with valve regurgitation (Figure 5). A corrugated tube replaced the ascending aorta and was sutured just above the aortic sinotubular junction (Figure 6). Around the junction area between the ascending aorta and the ventricular mass, we noticed cavitation with irregular margins, partially filled by liquefied material, of a yellowish-brown color (Figure 5). There was also a concave circular lesion of the aortic intima, with 1.5 cm in diameter, just below the emergence of the renal arteries. The lungs showed areas of wine-colored parenchyma condensation, triangular in shape when sectioned. There were signs of generalized visceral congestion, as well as ascites (700 mL) and bilateral pleural effusion (200mL in each hemithorax).

**Figure 7:**
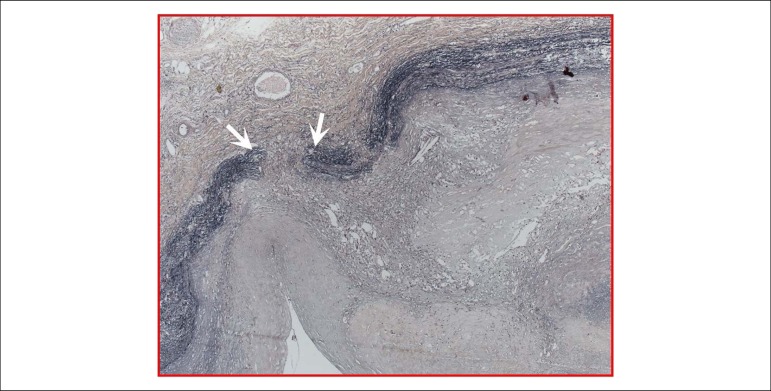
Photomicrography of the aortic wall at the level of the cavitary lesion described in the abdominal aorta. One can observe the rupture (between the arrows) of the elastic fibers (black bundles) of the tunica media, characterizing localized intramural dissection. Verhoeff staining for elastic fibers, objective magnification =5x.

**Figure 8:**
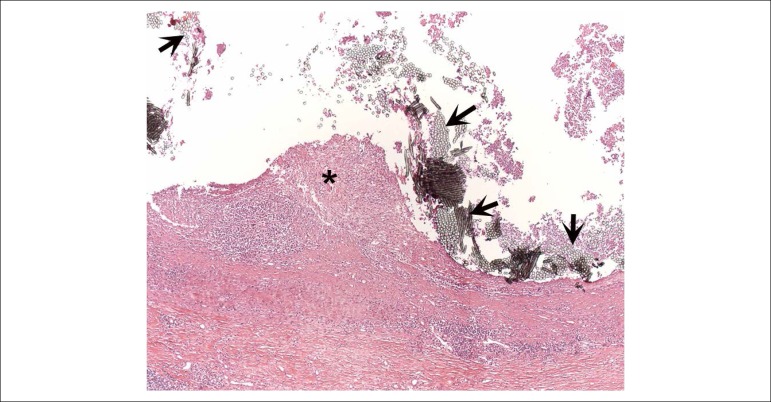
Photomicrography of the cavitary lesion wall described in the aortic root. We can see accumulations of polymorphonuclear neutrophils (asterisks) amid the synthetic tissue and sutures (arrows). Hematoxylin-eosin staining, objective magnification =10x.

Histological examination showed accumulation of mucoid material in the tunica media throughout the aorta, as well as focal rupture of elastic fibers in the cavitary lesion described in the abdominal aorta (Figure 7). The anatomopathological study of the peri-aortic cavitary lesion showed mixed inflammation, with necrotic cellular and polymorphonuclear neutrophil debris, among sutures and other synthetic materials (Figure 8). Bacterial and fungal tests were negative at this site. The aortic valve showed fibrous thickening of the margins. There was chronic passive visceral congestion, with hepatic centrolobular necrosis; the wine-colored lesions in the lungs corresponded to recent infarctions. **(Dr. Vera Demarchi Aiello)**

## Anatomopathological diagnoses

- Post-surgical correction of acute dissection of the ascending aorta
- Peri-aortic cavitary lesion, with mixed inflammatory reaction, without the identification of infectious agents
- Intramural dissection located in the abdominal aorta
- Aortic valve regurgitation
- Recent pulmonary infarctions

**Cause of death**: Congestive heart failure with terminal shock **(Dr. Vera Demarchi Aiello)**

## Comments

Aortic dissection is a serious disease, which is usually associated with systemic arterial hypertension and has as morphological finding the delamination of the vessel wall, with an intimal orifice called the "intimal tear" usually located in the ascending aorta, and the creation of a false lumen. This can extend to the tunica adventitia and undergo rupture, with massive bleeding into a cavity (pericardial, pleural or abdominal cavity).

When the dissection does not rupture, there is usually an orifice called a re-entry, located more distally in the aortic lumen, usually in the descending aorta.

Histologically, the presence of glycosaminoglycan accumulation in the tunica media, sometimes in the shape of the so-called "mucoid lakes",^10^ in addition to the rarefaction and fragmentation of elastic fibers and decrease of collagen in the external third of the aortic wall can be observed, leading to the weakness of this part of the wall.

In addition to the rupture, multiple organ ischemia due to the flow steal in the false lumen and aortic valve regurgitation are complications, due to collapse of its insertion when the dissection orifice is nearby.

In our case, the dissection was limited to the ascending aorta, which was replaced by a synthetic tube. Although there was a reference to aortic valvuloplasty in the surgery, the patient developed congestive heart failure, probably as a result of the remaining valvular regurgitation, which was not detected on the echocardiogram, possibly due to hemodynamic changes (patient in shock). This situation was responsible for the poor postoperative evolution.

The finding of a cavitary lesion in the aortic root, associated with the sutures, containing a purulent-like fluid, could mean local infection, but histological analysis did not detect the presence of microorganisms. **(Dr. Vera Demarchi Aiello)**
